# Global Transcriptomic Analysis of Interactions between *Pseudomonas aeruginosa* and Bacteriophage PaP3

**DOI:** 10.1038/srep19237

**Published:** 2016-01-11

**Authors:** Xia Zhao, Canhuang Chen, Wei Shen, Guangtao Huang, Shuai Le, Shuguang Lu, Ming Li, Yan Zhao, Jing Wang, Xiancai Rao, Gang Li, Mengyu Shen, Keke Guo, Yuhui Yang, Yinling Tan, Fuquan Hu

**Affiliations:** 1 Department of Microbiology, Third Military Medical University, Chongqing, 400038, China; 2 No. 180 Hospital of PLA, Quanzhou City, Fujian Province, 362000, China; 3 Institute of Burn Research, Southwest Hospital, Third Military Medical University, Chongqing, 400038, China

## Abstract

The interactions between Bacteriophage (phage) and host bacteria are widespread in nature and influences of phage replication on the host cells are complex and extensive. Here, we investigate genome-wide interactions of *Pseudomonas aeruginosa* (*P. aeruginosa)* and its temperate phage PaP3 at five time points during phage infection. Compared to the uninfected host, 38% (2160/5633) genes of phage-infected host were identified as differentially expressed genes (DEGs). Functional analysis of the repressed DEGs revealed infection-stage-dependent pathway communications. Based on gene co-expression analysis, most PaP3 middle genes were predicted to have negative impact on host transcriptional regulators. Sub-network enrichment analysis revealed that adjacent genes of PaP3 interacted with the same host genes and might possess similar functions. Finally, our results suggested that during the whole infection stage, the early genes of PaP3 had stronger regulatory role in host gene expression than middle and late genes, while the host genes involved amino acid metabolism were the most “vulnerable” targets of these phage genes. This work provides the basis for understanding survival mechanisms of parasites and host, and seeking phage gene products that could potentially be used in anti-bacterial infection.

Bacteriophage (phage) is a kind of virus that completes its replication cycle in the living bacteria. The replication cycle of phage refers to a period from penetration of a phage into a host cell to the release of progeny viral particles. One-step growth curve of phages is usually used to describe dynamic changes of phage replication^1^. At different stages of replication, phages express different gene products to direct the cellular enzymatic machinery to reproduce phage components^2^. Host DNA transcription, the first and most regulated step during gene expression, is commonly inhibited by phage during infection, which results in global changes of host cellular mRNA and proteins^3^. However, host bacteria undertake appropriate responses to control the replication of phages, such as immunity or resistance to phage infection^4^. Consequently, interactions between phages and host bacteria during one-step growth curve of phages become widespread and extensive. These interactions have driven the co-evolution of phage and host bacteria^2^. Therefore, knowledge of phage-host interactions is important for understanding the survival mechanisms of parasites and host cells.

Phage-host interactions have been studied for many decades^5^. However, prior to the current genomics era, it was nearly impossible to observe global genome-wide interactions of phages and their hosts. Advancements in genome sequencing and microarray technologies make it possible to observe phage-host interactions during phage replication process. Recently, two studies examined bacteria-phage interactions by microarray analysis were performed with only one or two experimental time points^6^,^7^. Two additional microarray analysis studies were designed to observe the bacterial host responses to phage infection over five time points, but these investigations did not assess bacteria-phage interactions^8^,^9^. The phages used in these studies were all strictly lytic phages, while few studies have addressed the interactions between a temperate phage and bacteria. Phage-host interactions are drastically altered when infected by virulent phages or temperate phages. *Escherichia coli* (*E. coli*) showed striking different response when infected by the temperate phage lambda and virulent phage T7. Fifty seven *E. coli* genes were identified to be in need for lambda phage replication, while eleven host genes were identified for the lytic-only infection by T7 phage^10^.

*P. aeruginosa* phage PaP3 is a temperate phage that was isolated and identified by our group and assigned to LUZ24–like virus. Genome sequencing revealed that PaP3 has a genome of 45503 bps with 71 ORFs (open reading frames) predicating as coding sequences (CDS)^11^,^12^. Specific functions have been assigned to less than 20% of PaP3 ORFs including most structural ORFs and those take part in DNA replication. However, the ORFs that were used to take over the host machinery have not yet been identified and the reproductive process of PaP3 in the host is still poorly understood.

We report here the first study, to our knowledge, of the genome-wide interactions between a temperate phage and *P. aeruginosa*. Transcriptomic profiles of both PaP3 and its host *P. aeruginosa* were observed with five infection time points by microarray analysis. In contrast to expression profiles of non-infection host, differentially expressed genes (DEGs) of phage-infected host were identified and then gene-interaction networks between PaP3 and host *P. aeruginosa* were constructed to reflect genome-wide interactions between PaP3 and host cells. According to functional analysis of DEGs induced by phage infection, the key phage-regulated pathways of *P. aeruginosa* were revealed at different stages of infection. These investigations shed light on the survival mechanism of phages and hosts, and exploring phage gene products that can control, even shut off, certain metabolism pathways of the hosts. These phage gene products may be potentially applied in anti-bacterial infection.

## Results

### One-step growth curve of PaP3

**Figure 1:**
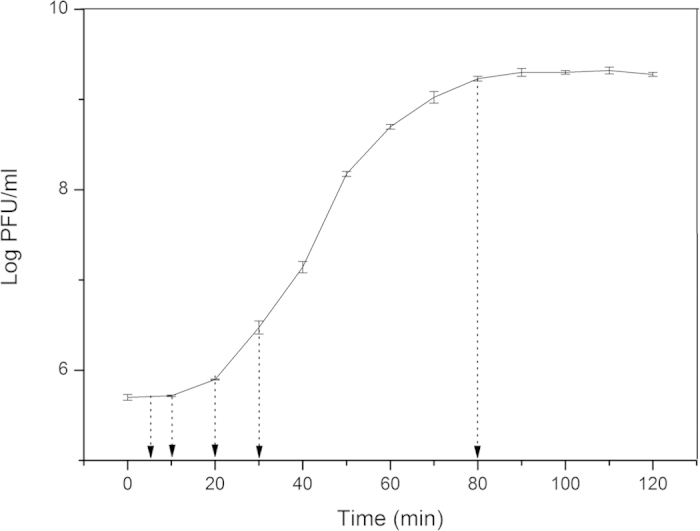
One-step growth curve of phage PaP3. Data were displayed as mean ± SD of three independent experiments.

Initially, the one-step growth curve of PaP3 in host bacteria *P. aeruginosa* was determined (Fig. 1). The results showed that replication cycle of PaP3 in host cells took about 80 min. The first 10 min was classified as early stage of PaP3 replication, with almost no release of progeny phages. This duration was the latent phase, where phages had just completed adsorption or injected their genome. The middle stage of PaP3 replication was 10–30 min. This stage was biosynthesis phase, wherein a number of phage components were produced. Late stage of PaP3 replication occurred at 30–80 min. In this duration, progeny phages were assembled and released from the host. The number of phage particles reached a peak at 80 min after phage infection, with a burst size of 31 phage particles per infected cell. Based on the one-step growth curve of PaP3, we set five time points (5 min, 10 min, 20 min, 30 min, and 80 min) in later experiments for observation of transcriptomic profiles of both PaP3 and host *P. aeruginosa*, with the phage-uninfected host cells as control.

### Temporal kinetic classes of PaP3 transcripts revealed by DNA microarray

**Figure 2:**
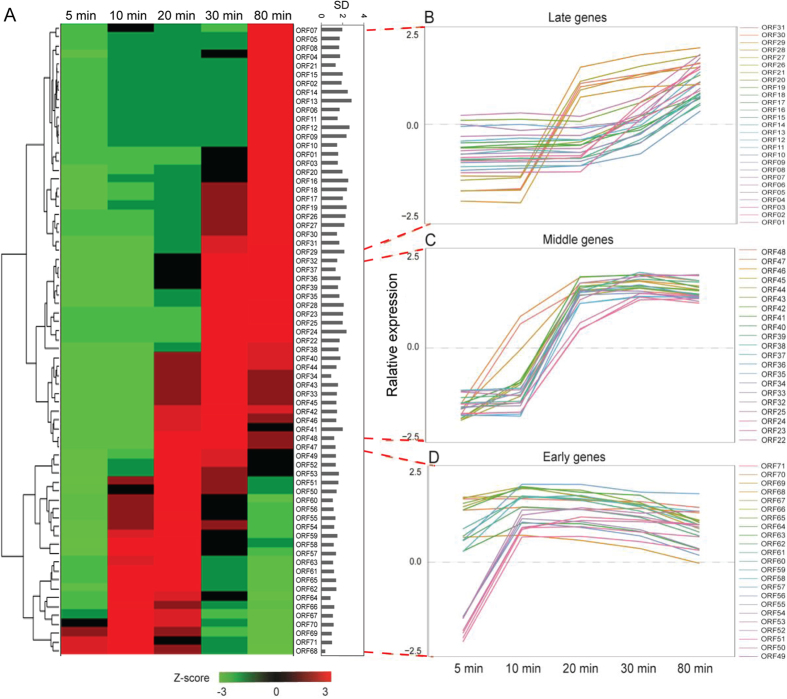
Temporal kinetic transcriptional profile of PaP3 genome. (**A**) Hierarchical cluster analysis of the expression data of PaP3 transcripts. The normalized expression levels across all the time points are color-coded: red for high and green for low expression levels. SD: the average value of standard deviation at different time points. (**B–D**) Seventy one ORFs of PaP3 divided into three temporal kinetic classes: early, middle, and late genes, also showing the time course of three transcript classes. The Y axis showed the normalized expression levels of each gene. The positive sign represented a higher expression level, while the negative sign represented a lower expression level.

A total of 71 ORFs of PaP3 were identified by DNA microarray analysis and the average fold change values for three independent biological replicates were calculated and normalized by the *P. aeruginosa* housekeeping gene *16S rRNA*. The results showed a significant differential expression pattern of all 71 ORFs (*p* < 0.01) from 5 min to 80 min post-infection. Cluster analysis of the expression pattern for differential expressed genes suggested that the 71 ORFs gathered into three temporal kinetic classes: early, middle, and late genes (Fig. 2A). Genes of ORF49–ORF71 were defined as early genes because of their expression values peaked at 5–10 min after phage infection. ORF22-25 and ORF32-48 showed the highest expression level at 10–30 min after phage infection and were defined as middle genes, while ORF1-21 and ORF26-31 were classified as late genes (Fig. 2B–D). According to the regulation of phage gene expression, the process of phage infection was also divided into three stages: early (5–10 min), middle (10–30 min), and late (30min–80 min).

**Figure 3:**
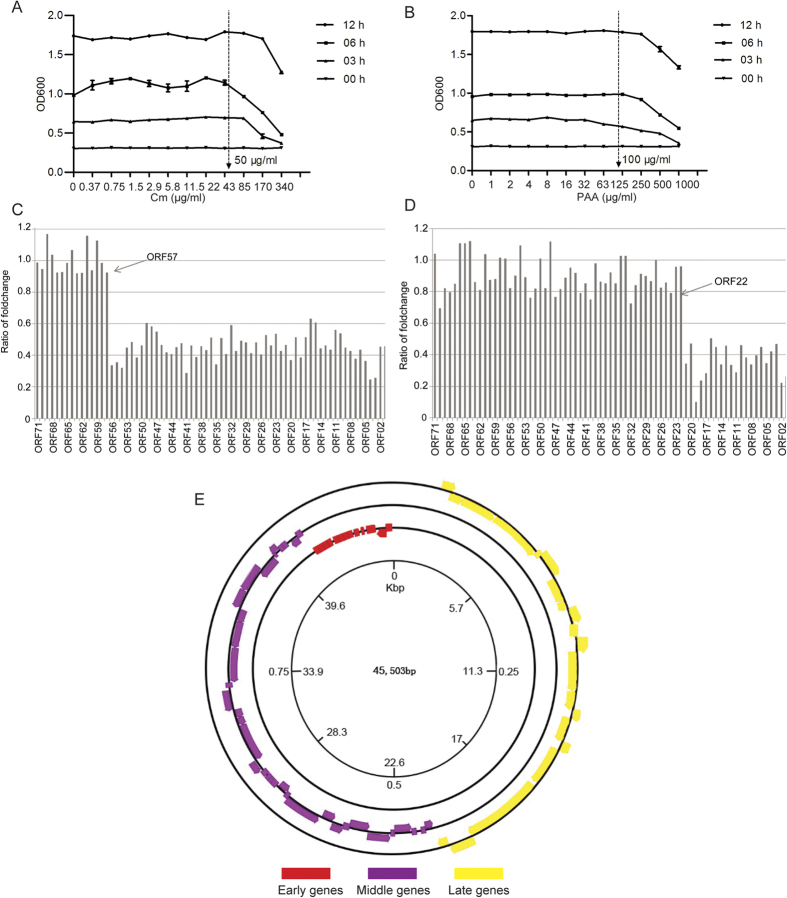
Validation for the temporal kinetic classes of PaP3 genes. (**A**,**B**) Drug inhibitor resistance experiment. The arrows indicate the optimum drug concentrations (50 μg/ml Cm or 100 μg/ml PAA) that did not affect the growth of the host bacteria and were used to verify the classification of PaP3 ORFs. (**C**,**D**) Expression patterns of PaP3 ORFs after treatment of Cm for 30 min and PAA for 80 min. (**E**) Cycle map of PaP3 expression transcriptional initiation was from the 5′ end of antisense strand and the early, middle were orderly expressed. The late genes were transcribed in initiation from 5′ end of sense strand.

The protein synthesis inhibitor cycloheximide (Cm)^13^ and DNA polymerase inhibitor phosphonoacetic acid (PAA)^14^ were added to bacterial-phage cultures to further validate the clustering result of PaP3 ORFs. From Fig. 3A and B, the maximum drug concentrations that did not affect the growth of host bacteria were 50 μg/ml for Cm and 100 μg/ml for PAA. Therefore, *P. aeruginosa* cultures under the optimal concentrations of Cm or PAA were infected with PaP3 respectively. Total RNAs were exacted after 30 min in the Cm experiment (Fig. 3C) or 80 min in the PAA experiment (Fig. 3D) for performance of microarray analysis. The expression of ORFs 57-71 were significantly up-regulated compared to ORFs 01-56, suggesting they were early genes because they are sensitive to the DNA polymerase inhibitor PAA but insensitive to protein inhibitor Cm. In contrast, the expression of ORFs 01-21 was inhibited by both Cm and PAA treatment and they were designated as late genes. Therefore, ORFs 22-56 between early and late genes were classified as middle genes.

### The global impact of PaP3 infection on host bacteria transcriptome

**Figure 4:**
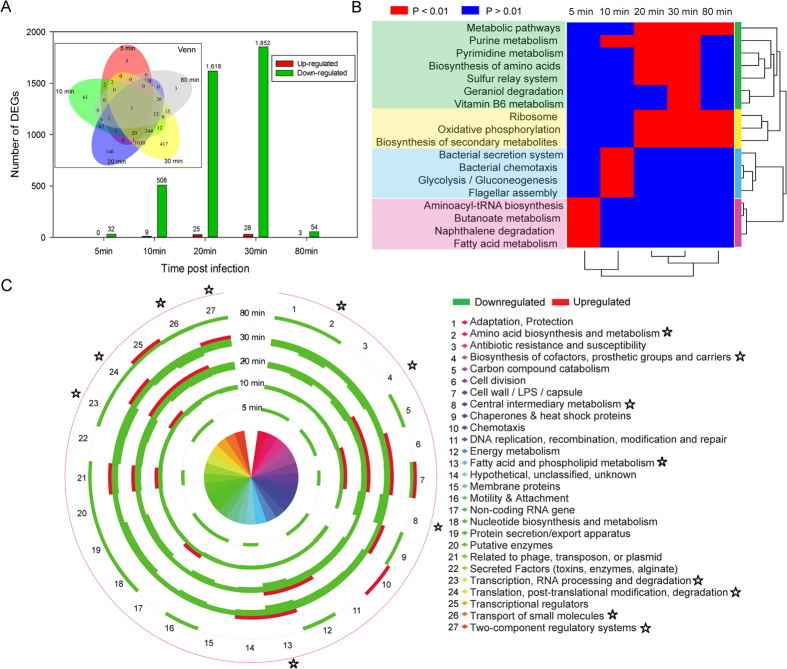
Analysis of differentially expressed genes (DEGs) of *P. aeruginosa* induced by phage infection. (**A**) The number and distribution of DEGs in different infection stages. The VennDiagram shows the intersection of the number of DEGs at each time point. (**B**) Infection-stage-dependent clusters of KEGG pathway map. The *p* values were calculated to show the significance according to the number and expression of DEGs genes in each pathway. Eighteen significantly changed KEGG pathways were indicated by red (*p* < 0.01) or blue (*p* > 0.01) at different time points. Hierarchical clustering analysis grouped the pathways into 4 clusters. (**C**) Comparison of PseudoCAP functional classification of the DEGs of *P. aeruginosa* at different infection time points. All of these data represent the percentage of the total number of DEGs in each functional class (Table S2). Down-regulated genes are shown as green and up-regulated genes shown as red. The pentacles indicate first eight function categories with a high proportion of down-regulated genes at 20 and 30 min.

To investigate the transcriptional response of *P. aeruginosa* to PaP3 infection, total cellular RNA was extracted from host cultures uninfected or infected with PaP3 for 5, 10, 20, 30, or 80 min, respectively. The purified samples were applied to perform DNA microarray. A total of 5,633 specific probes were designed for analysis of host gene expression. Globally, there were 38% (2160/5633) DEGs of *P. aeruginosa* comparing the infected samples to the uninfected samples (Fold change > 2, *q* < 0.05); and 98% (2120/2160) DEGs were down-regulated (2120 down-regulated genes and 40 up-related genes). It was worth mention that the number of DEGs per time point did not sum up to the total DEGs number 2160 but 4129, which was due to the same gene affected at different time points (Fig.4A). However, there was no interpretation between up-regulated and down-regulated genes. Three stages of PaP3 infection were classified according to the replication cycle of PaP3: early (5–10 min), middle (10–30 min), and late (30–80 min) stage. The number and distribution of DEGs cross different infection stage suggested that the inhibited genes were mainly in middle stage of infection (10–30 min) (Fig. 4A). A total of 1852 genes were most abundantly inhibited at 30 min after phage infection and the largest number of up-regulated genes (28) also appeared at this time point. Combining these results with the one-step curve of PaP3 (Fig. 1) suggested that the impact of PaP3 on host *P. aeruginosa* gene expression was mainly in biosynthesis phase of phage.

### PaP3 infection induces infection-stage-dependent changes of pathways and biological functions in *P. aeruginosa*

All the 2160 DEGs were distributed into 112 KEGG (Kyoto Encyclopedia of Genes and Genomes) pathways^15^. Based on the calculated *p*-value of each KEGG pathway by Fisher Exact Test at each time point, 18 pathways were significantly changed (*p* < 0.01) at least one time point (Table S1). All genes except for PA0631 and PA3589 in the 18 pathways were down-regulated genes. Both PA0631 and PA3589 participated in the pathway of fatty acid metabolism. From the time distribution of pathways that were inhibited significantly, 5 min and 10 min showed the most different pathway category and 20 min showed similar trends as 30min, while 80 min had some overlaps with the middle infectious stage (Fig. 4B). Hierarchical clustering analysis grouped 18 pathways into four infection-stage-dependent clusters. At 5 min after PaP3 infection, the most regulated pathways were related to the carbon source of bacteria including naphthalene degradation, pyruvate, and butanoate metabolism. The motility of the host such as chemotaxis and flagella assembly was significantly affected at 10 min after infection. The most extensively changed KEGG pathways were seen at the 20 min and 30 min infection stage, which involved biosynthesis of secondary metabolites, geraniol degradation, and biosynthesis of amino acids, of which, geraniol degradation and vitamin B6 metabolism were the most affected pathways (Table S1).

Of these pathways, the category of ribosome was significantly down-regulated over a long infection period (20–80 min) with *p* values ranging from 1.6E-08 to 2.2E-16 (Table S1). The ribosome pathway contained 70 genes encoding ribosomal RNA (rRNA) and ribosomal protein (rProtein, including large subunit and small subunit) and an average of 43 rProtein genes were inhibited at 20–80 min post-infection. The oxidative phosphorylation of host was also significantly disturbed with *p* values ranging from 1.1E-05 to 3.1E-11 at middle and late stage of PaP3 infection. These down-regulated genes were related to multiple oxidative phosphorylation enzyme complexes including NADH dehydrogenase, F-type ATPase, succinate dehydrogenase, cy*tochrome c* reductase and *cytochrome c* oxidase.

To integrate complementary information from different databases and gain complete insight into the functions of DEGs, the microarray data were also classified according to the categories described in the *Pseudomonas aeruginosa* Community Annotation Project (PseudoCAP)^16^. As shown in Fig. 4C, the DEGs were classified into 27 PseudoCAP functions and the percentage of the total number of DEGs in each functional class was calculated to show the significant enrichment functional terms (Table S2). The genes involved in translation, post-translational modification, degradation and carbon compound catabolism were inhibited at 5 min and 10 min. The genes of cell division were not affected by phage infection at 5 min, but were then down-regulated from 10 to 30 min. The distribution of DEGs at 20 and 30 min showed the approximate profiles, which disclosed that 8 functional categories (indicated by pentacles) were obviously repressed (ratio > 40%). The first three functional terms with larger ratio values at both 20 and 30 min were fatty acid and phospholipid metabolism, transport of small molecules and amino acid biosynthesis and metabolism.

### Biological functions of *P. aeruginosa* induced by PaP3 infection

Our microarray data identified only 40 up-regulated genes (Fold change > 2, *p* < 0.05). No DEGs were identified at 5 min after phage infection. These genes related to 14 PseudoCAP functions (Fig. 4C) and the most functions reduced by phage were observed at 20 min with 9 terms. The functions related to cell wall/LPS/capsule, translation, post-translational modification, degradation and related to phage, transposon, or plasmid were induced during middle stage of phage infection. The results suggested that the reproduction of PaP3 highly demanded the translation machinery of host cells. Among these up-regulated genes, PA0908 encoded a hypothetical protein involved in the cellular response to antibiotic according to Gene Ontology (GO) analysis, suggesting that this protein was probably related to bacterial positive response to phage infection.

### RT-qPCR validation of selected DEGs

**Figure 5:**
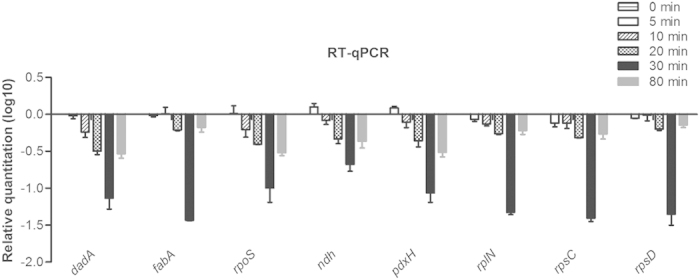
RT-qPCR validation of selected DEGs. Eight inhibited genes of *P. aeruginosa* by PaP3 infection were selected from microarray data set. The qPCR results were normalized using *16S rRNA* and expressed as fold change (Log_10_ scale) by the comparative Ct method. Control (0 min) is normalized as 0.

Real-Time qPCR were performed to validate eight DEGs at various infection time points (Fig. 5). We focus on three genes (*dadA*, *fabA* and *rpoS*) that were down regulated during the whole infection progress. The RT-qPCR results showed a consistent directional change compared to microarray assay. Furthermore, five other genes related to the inhibited KEGG pathways (Fig. 4B) at the middle stage of infection were analyzed by RT-qPCR. Three pathways were involved: oxidative phosphorylation (*ndh*), vitamin B6 metabolism (*pdxH*) and ribosome (*rplN*, *rpsC* and *rpsD*). According to the RT-qPCR results, all of them were down-regulated drastically during 10–30 min post infection. In addition, all these genes were observed most severely down-regulated at 30 min after phage infection, which was in line with the distribution of DEGs that the most DEGs were identified at 30 min post infection (Fig. 4A).

### Gene co-expression analysis reveals negative correlations between phage and host transcriptional regulators

Next, we examined how PaP3 that only harbors dozens of genes shut off thousands of host genes. We turned our attention to the bacterial transcriptional regulators (TRs), which regulates lots of downstream genes globally. We hypothesized that specific gene products of PaP3 might target host TRs and lead to differentially expressed downstream genes. Gene co-expression analysis for phage and host TRs was performed on the basis of microarray data from middle infection stage (10–30 min) groups that including the most DEGs (Table S3). Generally, genes with positive correlation (cor > 0) tend to share similar function, while negative correlation (cor < 0) indicated inhibitory actions^17^. In the co-expression analysis, 24 pairs of genes with negative correlation and only one pair (ORF52-PA0163) with positive correlation was identified (*p* < 0.01, |cor| > 0.99). These results suggested the inhibitory action of phage to many host TRs and confirmed our hypothesis. Eighteen out of the 19 PaP3 genes were middle genes, which were generally considered as DNA-replicating genes. Among the 13 TRs genes, only four were annotated specific functions: *argR*, *gecA*, *rpoS*, and *vqsR*. It is worth noting that ORF53 was observed to be negatively correlated with three TRs (*argR*, *gecA*, and *vqsR*) regulating bacterial quorum sensing (QS) system, biofilm and virulence^18^,^19^,^20^,^21^. So it could be inferred ORF53 might repress bacterial virulence globally and could potentially be studied further as an anti-bacterial agent candidate.

### Gene interaction networks between PaP3 and host at each stage of infection

**Figure 6:**
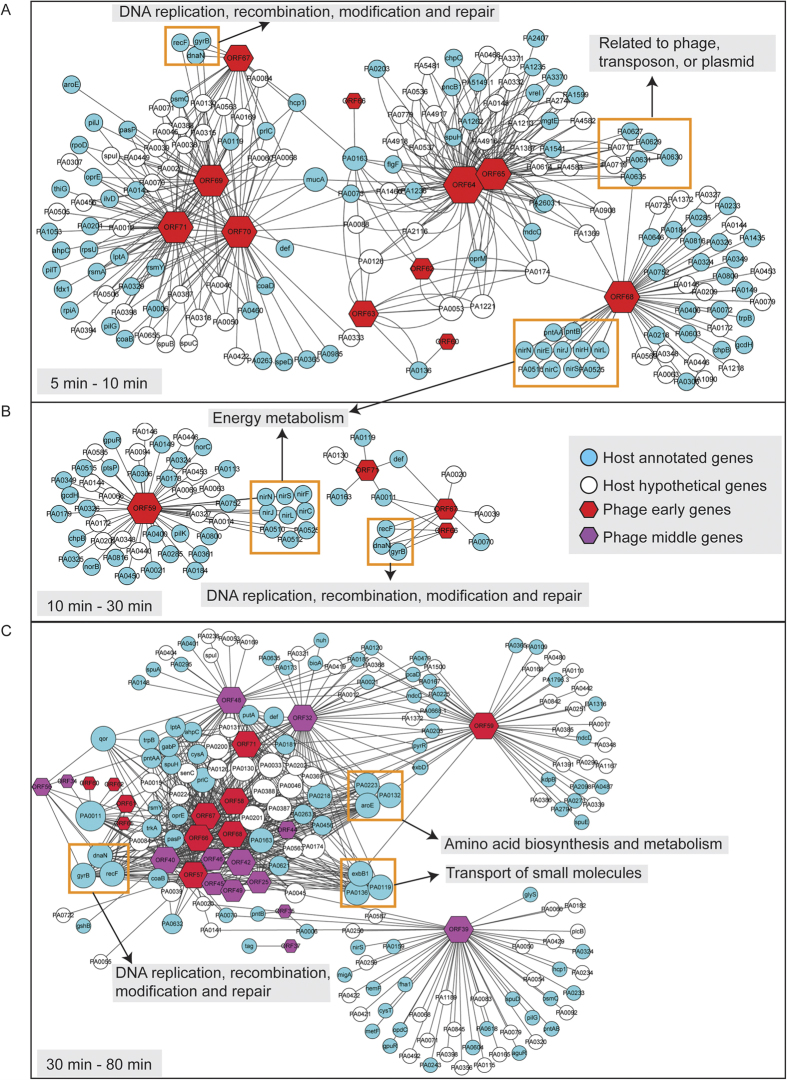
Gene co-expression network between phage PaP3 and host at early, middle and late stage of infection. The hexagons represent phage genes and circles the host genes. Two colors of nodes represent the early (red), middle (purple) PaP3 genes, respectively; the blue and white nodes are respectively the annotated or hypothetical genes of host bacteria. Extensive level of interaction between PaP3 and *P. aeruginosa* genes was quantified by K-score range from 1 to 5 (Table S5). Genes with a higher K-score (more links) are shown in bigger size.

To track the biological function of a specific phage gene at a specific stage of infection, three networks were constructed for different stages based on gene co-expression analysis (Fig. 6). Genes in the networks were all positive correlations when *p* < 0.01 and |cor| > 0.99 (Table S4). This meant that the genes that appeared in one sub-network might share similar biological processes. Extensive level of interaction between PaP3 and *P. aeruginosa* genes was quantified by K-score range from 1 to 5 (Table S5). Genes with higher K-scores (more links) were shown in bigger size and could be considered as regulatory genes. Remarkably, ORF68 and ORF59 were showed to be involved in bacterial operons of energy metabolism including operon *nir* and *nor* encoding enzymes of denitrification and operon *pntAA-pntB* mediating the energy-dependent reduction of NADP^+^ with NADH. This finding suggested that ORF68 and ORF59 could be used to control cellular energy metabolism. Three middle genes of PaP3 had been assigned with the function of DNA replication: ORF32 (DNA polymerase 1), ORF39 (DNA polymerase 2), and ORF40 (Primase/helicase). Three host genes (*dnaN*, *gyrB*, *recF*) in the network were related to DNA replication, recombination, modification, and repair, which were positively correlated with ORF32, ORF39, and ORF40 that were related to DNA replication. This result confirmed the feasibility of using gene co-expression to predict unknown gene biological function. Therefore, we predicted ORF67 had a key role in regulating DNA replicating and repair, since it was positively correlated with *dnaN*, *gyrB*, and *recF* in all the stages (Fig. 6A–C). BlastX (search protein database using a translated nucleotide query) was performed to search for evidence of the putative functions of ORF68, ORF59, and ORF67. The results showed that there were certain similarities of ORF68 and ORF59 with the enzymes related to energy metabolism: the chain A of phosphoglycerate oxidoreductase from *Vibrio Cholerase* O395 (Identities = 30%) or GTPase Der from *Enterobacter sp*. 638 (Identities = 38%), respectively. While ORF67 showed similarity with the chain A of human topoisomerase I DNase complex (Identities = 67%) that were indeed involved in DNA replication and repair.

Except for these specific functional cluster, majority of the phage genes were showed to be linked with multiple host functions. It meant that multiple biological functions could be carried by one phage gene and it was an advantage for phage to control the expression of the huge host genome. An interesting effect was observed that the phage genes clustered in a same sub-network tended to be clustered along DNA such as ORF64-65, ORF66-68, and ORF69-71. The host genes with high K-scores appeared at late network, which could be hotspots specific to the regulation of phage (Fig. 6C). These hotspots were related to the function of amino acid biosynthesis and metabolism, DNA replication, recombination, modification and repair as well as transport of small molecules.

### PaP3 early genes play key regulatory roles in bacterial amino acid biosynthesis and metabolism

**Figure 7:**
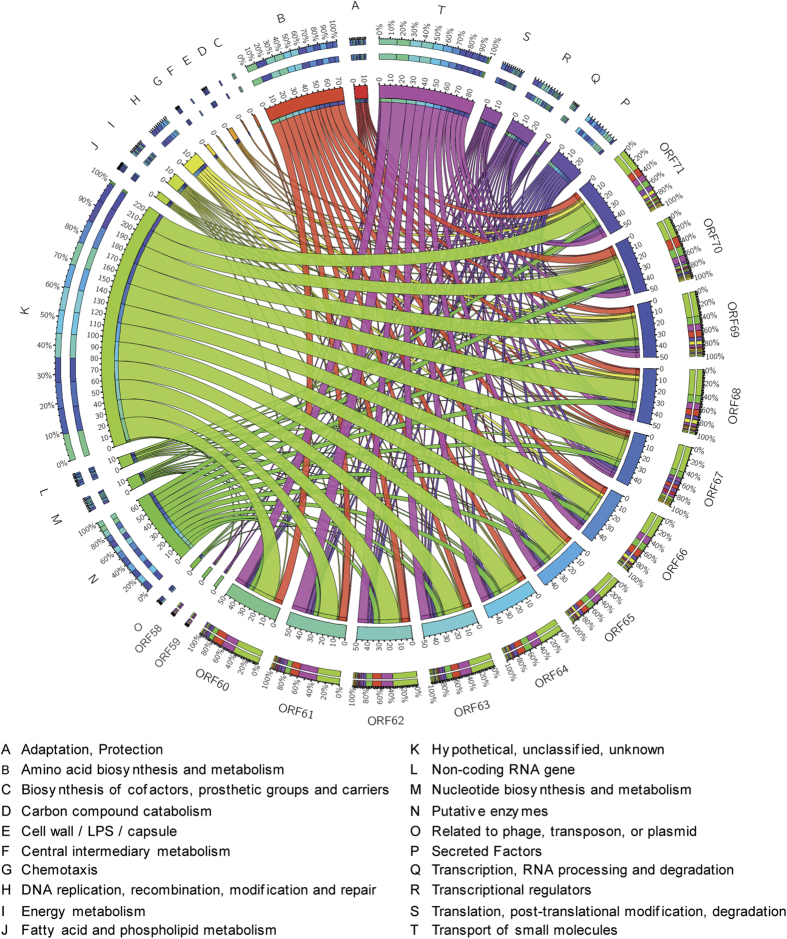
Merged gene co-expressed network between phage and host. The circos plot was constructed by integrating gene co-expression network from all the time points, which consisted of 20 PseudoCAP functions of host and 14 ORFs of PaP3. The number of host target genes of each phage gene in each PseudoCAP function term was calculated to show the biological functions of host linked with phage genes. The PseudoCAP functions were shown with different color and the number of host genes in each functional term was indicated with different size of link. Wider links meant the more host genes in a PseudoCAP function term. The structure of the color box for phage genes (the outer-most ring) indicated the functional composition of their host gene partners.

In order to investigate the overall interactions between phage and host during all the stages of infection, a merged network of gene co-expression with five time points was constructed by circos^22^. According to the gene expression level at each time point, a total of 110 host genes and 14 early PaP3 genes were screened out and all the correlations were positive when *p* < 0.01 and |cor| > 0.99 (Table S6). There were no middle or late genes of PaP3 showed significantly correlation with host gene, which suggested the key regulatory roles of PaP3 early genes during the whole infection period. The number of target genes of each phage gene in each PseudoCAP function term was calculated to show the biological functions of host linked with phage genes. Finally the merged network was constructed with 20 PseudoCAP functions and 14 ORFs of PaP3 (Fig. 7). Except for the functions of “hypothetical, unclassified, unknown” and “putative enzymes,” the terms of “transport of small molecules” and “amino acid biosynthesis and metabolism” were showed to be linked with more phage genes. ClueGO/CluePedia plugin of Cytoscape software^23^,^24^ was used to analyze the KEGG pathways of the 110 host genes included in this network at K-score > 0.4 and *p* < 0.05. The result showed that amino acid metabolism was the most abundant pathway. These metabolism pathways were related to various amino acids including arginine, proline, phenylalanine, and among others (Fig. S1). As previously mentioned, adjacent genes of PaP3 probably acted with similar function. In this network, ORF60-63 were observed to interact with same function of the host (similar structure of color box), and so did ORF64-65 and ORF68-69.

## Discussion

The intracellular phage-host interaction changes with the phage development, which causes differential gene expression profiles. In this study, the dynamic interactions between *P. aeruginosa* and *P. aeruginosa* phage (PaP3) were investigated using time-series phage-infected samples. First, the kinetic class of PaP3 genome was generated by cluster analysis and Cm/PAA inhibition experiments. There were 15 ORFs (ORF57-71) which were classified as early genes, 35 ORFs (ORF22-56) as middle genes and 21 ORFs (ORF1-21) were late genes. PaP3 was classified as a member of LUZ24-like phages. Comparing the temporal transcriptional map of PaP3 and LUZ24 revealed that genes with high similarity were distributed in the same phase (Fig. S2). This further confirmed our classification of PaP3 genes.

The investigation of host cell transcriptional response to phage infection has been a vigorous area of transcriptome research by microarray or RNA-seq in recent years. In our study, a large number of phage-induced DEGs of *P. aeruginosa* were observed *via* microarray analysis. At 5 min after infection, phage early genes were slightly expressed shortly after viral DNA entered the cell and had less impact on the host. It was not until 10 min after infection that all the phage early genes were expressed relative to the peak expression value, and then phage products began to hijack the cellular resources with an increasing number of host DEGs. A phage–host balance was achieved in the culture wherein most bacteria were lysed but existed as phage-resistant mutants or the bacteria containing lysogenic phage at late stage of infection (80 min).

Phages require a host organism to propagate, which involves manipulation of multiple host proteins, molecular processes, cellular pathways that are related to transcription and translation, signal transduction and metabolism. Given that phages infect specific hosts by their binding specifically to certain receptors, several studies showed phages also attack specific intracellular pathways on the basis of the interactions with host proteins. The early protein Gp0.4 of phage T7 could directly inhibit the *E. coli* filamenting temperature-sensitive mutant Z division protein, and confer a competitive advantage to phage T7 by inhibiting cell division^25^. Another analysis on the interactions between phage c2 and *Lactococcus lactis* clearly showed that the phage infection induced the molecular mechanisms involved in the host response to the membrane perturbations^7^. PseudoCAP function analysis in our work also indicated the early DEGs participated in the “cell wall/LPS/capsule” function.

Clustering of significantly changed KEGG pathways revealed interesting infection-stage-dependent pathway communications that the bacterial pathways related to carbon source and motility were inhibited at early stage of infection, while protein syntheses and energy metabolism were respectively disturbed at the middle and late stages. The earliest significantly changed pathway was related to the carbon source, such as the pyruvate metabolism. A previous study showed that the viral adsorption and penetration at the early stage can lead to stimulation of pyruvate metabolism in phage-infected cells because of the reduced rate of oxidation of pyruvate^26^. In addition, carbon source metabolism provides fuel for the rapid growth of bacteria and plays a critical role in the control of cell death. The cell division was not affected by PaP3 infection at 5 min post infection, while the genes related to cell division were not changed during the PRD1 infections of 0–50 min^9^. PaP3 showed strong inhibition to the host energy metabolism by down-regulating massive genes involved in enzymes of respiratory chain, which was a common phenomenon in other phage infection such as BtCS33 and lambda phage^27^,^28^. Host protein synthesis was also strongly depressed by PaP3 by the reduction of ribosome. It was known that phage infection caused the shutoff of host macromolecular synthesis (DNA, RNA and proteins), which might be the result of lack of energy supply^29^.

Genes in the same pathway, functional complex, regulatory network, and signaling circuits often exhibit similar expression patterns^30^. According to PseudoCAP function, there were 476 transcriptional regulators (TRs) in *P. aeruginosa*. TRs were used to regulate downstream genes in response to the change of environmental and physiological conditions by DNA-binding. For bacteriophage, TRs are a beneficial shortcut to manipulate host genes to stimulate specific transcriptional circuits. Phage genes are expressed sequentially by use of the host transcription mechanism. This process was realized by modifying host RNA polymerase or interfering TRs that recognize regulatory elements^31^. Many studies have showed that phage directed host transcription by interacting with regulators to shut off host transcription and cause the sequential activation or inactivation of different phage promoter classes (early, middle and late)^32^,^33^,^34^. The early genes of phages usually encode regulatory proteins that modulate expression of phage and host genes, which were most likely to correlate with host transcriptional regulators. However, there were no early genes of PaP3 identified to interact with host TRs at middle stage of infection. This could be that the early phage regulatory products took effect at early stage of infection. In fact, only one pair of genes (ORF65 and *rpoS*) of significant correlation was observed in the 5 min (*p* = 0.0027, cor = −0.9945). We speculate that ORF65 and *rpoS* (encoding the stress sigma factor RpoS of *P. aeruginosa*) play a regulatory role in opposite directions and ORF65 would probably inhibit the function of RpoS. Our results showed negative interactions between phage middle genes with host TRs regulating bacterial quorum sensing system, biofilm, and virulence. Bacterial quorum sensing system, biofilm, and virulence may represent a class of anti-bacterial targets and were the breakthrough in anti-bacterial therapy^35^,^36^. The PaP3 genes that correlated with bacterial global regulators of these anti-bacterial targets would be further studied as candidate anti-bacterial factors.

The gene co-expression network analysis is also a powerful tool to extract function-related genes, identify novel genes and their biological functions^37^,^38^. In recent years, gene co-expression analysis has been widely used for predicting unknown gene biological function^39^. However, to our knowledge, this is the first study that infers biological functions of phage genes based on the gene co-expression network between phage and host. The networks indicated that phage genes regulated host genome through a complicated and highly modulated gene network. The late genes of PaP3 encoding structural proteins were not observed in the network, which suggested the weak regulatory role of the phage late genes.

Although host-phage interactions are involved in all stages of the infection cycle, a survey has revealed that most (64%) of these interactions involve phage early proteins^40^. Moreover, the phage early genes within our merged network showed a strong relationship with bacterial amino acid metabolism. Since amino acid acts as energy source for bacterium, the depletion of the intracellular supply of free amino acids causes the limitation of bacterial growth. For phage, massive replication also required abundant amino acid resource. The specific protein interactions of phage with its host may alter the products of amino acid metabolism or amino acid pool to serve the needs of phage multiplication. In addition, we predict that the earlier inhibition of bacterial amino acid metabolism by early protein improves the efficiency in utilizing cellular resources.

Phage early genes were considered as anti-bacterial candidates in a growing body of research. However, majority of these phage genes have not been assigned a biological function. The functional characterization of these early proteins is one of the major challenges for phage biomedical research. Therefore, the work presented herein provides a strategy for characterization of unknown early phage proteins. Since phages employ various strategies to attack host bacteria, the systematic study on phage-host interactions will contribute to our knowledge about phage life cycles and have the potential to reveal important or novel bacteria-phage interactions that could be used for microbial drug therapy.

## Materials and Methods

### Bacterial strains and growth conditions

*Pseudomonas aeruginosa* PA3 and *Pseudomonas aeruginosa* phage (PaP3) were stocked in our laboratory. *P. aeruginosa* was cultured in Luria-Bertani (LB) broth medium aerobically at 37 °C for all experiments. PaP3 particles were collected and purified using CsCl gradient ultracentrifugation.

### One-step growth curve

For the one-step growth curve of PaP3, we used a modification of the methods of Lu *et al*.^1^. The early logarithmic growth phase cultures of *Pseudomonas aeruginosa* (OD600 = 0.2) were continuously diluted 10 times. Bacteria cells were calculated by CFU plate counts. Phage samples were added to 3 ml PA3 cultures (OD600 = 0.1) at multiplicity of infection (MOI) of 10. After incubation at 37 °C for 15 min to allow the adsorption of phage, the mixture was then centrifuged for 30 s at 13,000 g. The supernatant containing any un-adsorbed phages was removed by washing twice with LB medium. Pellets were re-suspended in 3 ml LB and the cultures were then grown at 37 °C, 180 rpm. Fifty microliters of samples were taken every 10 min, and the number of PaP3 particles was determined by the double-layer agar plaque method. The burst time and burst size were calculated based on the one-step growth curve.

### RNA extraction and microarray analysis

Total RNA was isolated from 6 groups (0, 5, 10, 20, 30, and 80 min post-infection) of bacterial culture that infected with PaP3 at MOI of 10 by SV Total RNA Isolation System (Promega) according to the manufacturer’s instructions. The uninfected samples (0 min) served as reference. The extracted RNA was utilized to microarray analysis and RT-qPCR.

For microarray analysis, three biological replicate RNA samples were taken at each time point. Microarray hybridization was performed by the Shanghai Biotechnology Corporation (http://www.shanghaibiotech.com/) according to the Agilent standard protocols. A total of 5633 probes were designed for *P. aeruginosa* genome including 5557 protein genes, 13 rRNA genes and 63 tRNA genes, while 314 probes (2–16 probes per ORF) for 71 ORFs of PaP3. Data analysis was conducted using microarray analysis software Gene spring GX10.0. Differentially expressed genes (DEGs; exhibiting at least two fold changes in expression) were screened *via* one-way ANOVA (*p* < 0.05). The associated *p* values were adjusted for multiple testing with the Benjamini and Hochberg method^41^ to keep the false discovery rate less than 5%. All the DEGs were listed in Table S8. The raw data of the microarray experiments are available in the GEO database (http://www.ncbi.nlm.nih.gov/geo) with an accession number (GSE68584).

### Real-time quantitative PCR

To validate microarray data, real-time quantitative PCR (RT-qPCR) was performed on 8 selected DEGs. Primers for RT-qPCR are listed in supplementary Table S7. The cDNA was synthesized from 1 μg of the total RNA by RevertAid™ first strand cDNA synthesis (Thermo Scientific, USA). The RT-qPCR reactions were performed using Viia 7 Real Time PCR system (Applied Biosystems) with Fast Start Universal SYBR Green Master (Roche). All samples from 3 biological replicates of each time point were amplified in a 384-well plate. The relative expression levels were normalized to the expression of *16S rRNA*.

### Cm and PAA inhibition experiment

Chloramphenicol (Cm) can prevent *de novo* protein synthesis of virus by preventing translation, and phosphonoacetic acid (PAA) can act as an inhibitor of DNA polymerase. Consequently, Cm allows expression only of early genes, while PAA inhibits expression only of late genes. To verify the PaP3 expression profile observed in the microarray experiments, the bacteria were treated with either Cm or PAA as previously described with modifications^13^,^14^. Briefly, 990 μl PA3 cultures (OD600 = 0.3) were added with 10 ul Cm (final concentration, 0.37–340 μg/ml) or PAA (final concentration, 2–1000 μg/ml). The OD600 was detected after incubation at 37 °C with constant shaking for 3, 6, and 12 hours, respectively. Then the suitable drugs concentration that did not affect the growth of bacteria was determined. During phage infection experiments, 5 ml PA3 cultures (OD600 = 0.3) were incubated in LB media with Cm (final concentration of 50 μg/ml) or PAA (final concentration of 100 μg/ml) and incubated at 37 °C for 5 min. A 1ml aliquot of cultures was used as control group, while the rest of the culture was infected with PaP3 particles at MOI of 10. After incubation for 30 min in the case of Cm and 80min for PAA, total RNA was isolated from each culture for microarray analysis.

### Construction of co-expression network

We employed a linear regression analysis to quantify the co-expression of interaction partners based on gene expression similarity of microarray data sets. The linear correlation coefficient (Pearson’s correlation coefficient, cor) was first computed as described previously^42^. To make a visual representation, only the strongest correlations (*p* < 0.01, |cor| > 0.99) between phage and host genes were selected to construct the network by Cytoscape^17^. Circos was used to draw the graph of the merged network for gene co-expressed analysis^22^.

## Additional Information

**How to cite this article**: Zhao, X. *et al*. Global Transcriptomic Analysis of Interactions between *Pseudomonas aeruginosa* and Bacteriophage PaP3. *Sci. Rep*. **6**, 19237; doi: 10.1038/srep19237 (2016).

## Supplementary Material

Supplementary Information

Supplementary Table S4

Supplementary Table S5

Supplementary Table S6

Supplementary Table S8
