# Supplementary material for: Global Transcriptomic Analysis of Interactions between Pseudomonas aeruginosa and Bacteriophage PaP3
Source: Sci Rep. 2016 Jan 11;6:19237. doi: 10.1038/srep19237 (PMC4707531; doi:10.1038/srep19237)
Supplement: Supplementary Information [file srep19237-s1.pdf]

Global Transcriptomic Analysis of Interactions between *Pseudomonas aeruginosa* and Bacteriophage PaP3

Xia Zhao[1], Canhuang Chen[2], Wei Shen[1], Guangtao Huang[3], Shuai Le[1], Shuguang Lu[1], Ming Li[1], Yan Zhao[1], Jing Wang[1], Xiancai Rao[1], Gang Li[1], Mengyu Shen[1], Keke Guo[1], Yuhui Yang[1],Yinling Tan[1,*], Fuquan Hu[1,*]

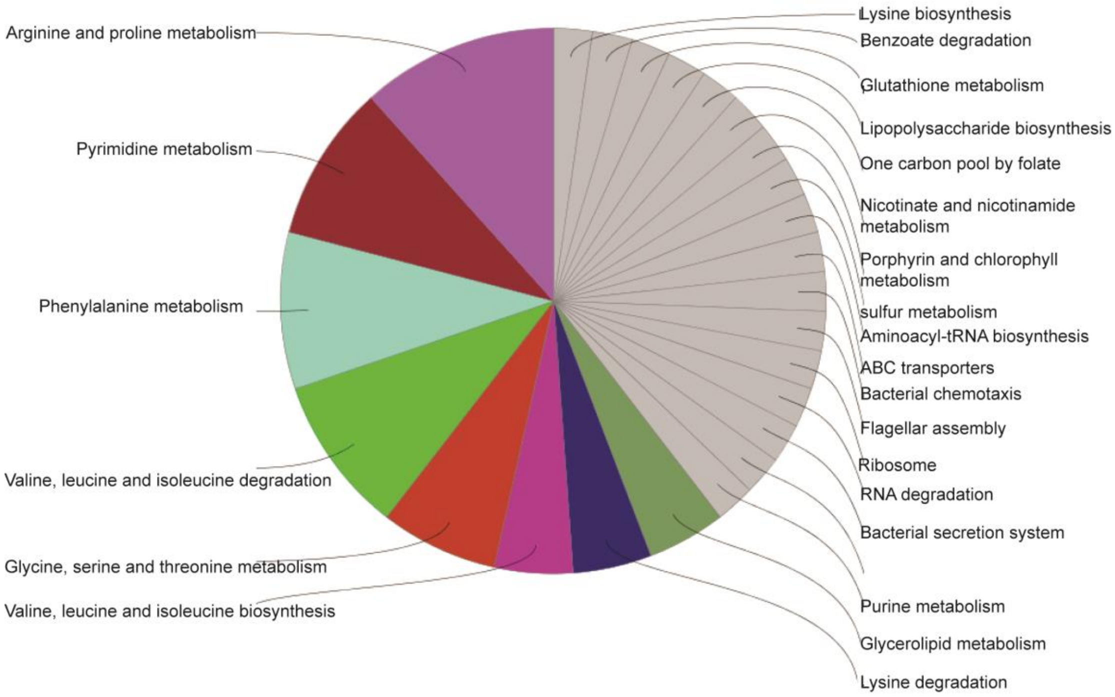

Figure S1. Enriched KEGG pathways of 110 host genes in the merged network. All 110 genes of *P. aeruginosa* in the merged network were grouped into 24 KEGG pathways (K-score > 0.4, P < 0.05). Data visualization was performed by ClueGO/CluePedia plugins of Cytoscape software.

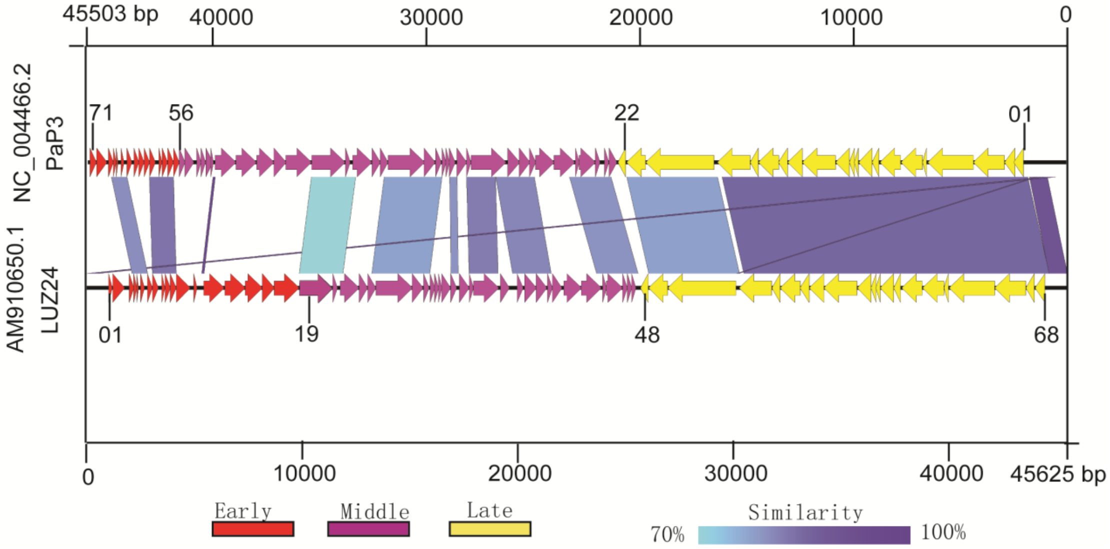

Figure S2. Genomic comparison of phage PaP3 and LUZ24. Putative ORFs are shown with arrows marked with the different color: early genes (red), middle genes (purple) and late genes (yellow). Boxes with different shades of blue represent degrees of similarities between genomes.

Table S1 The p-value of significantly changed KEGG pathways.

KEGG pathway	5 min	10 min	20 min	30 min	80 min
Aminoacyl-tRNA biosynthesis	0.002847	0.0381	0.01103	0.07519	0.8436
Bacterial chemotaxis	0.1818	0.004459	0.2288	0.452	0.7706
Bacterial secretion system	1	0.001407	0.001041	0.002264	0.6869
Biosynthesis of amino acids	0.5637	0.05282	8.04E-08	1.55E-07	0.1147
Biosynthesis of secondary metabolites	0.8472	0.1228	5.41E-05	3.8E-05	0.00452
Butanoate metabolism	0.003654	0.7211	0.4368	0.2782	0.1253
Fatty acid metabolism	0.009829	0.4029	0.5599	0.3825	0.02331
Flagellar assembly	0.1271	0.000191	0.03886	0.08688	0.1795
Geraniol degradation	0.4727	0.2349	0.01203	0.000248	0.6948
Glycolysis / Gluconeogenesis	0.07829	0.006753	0.01245	0.0508	0.3593
Metabolic pathways	0.8949	0.2031	2.47E-08	3.81E-13	0.001815
Naphthalene degradation	0.008149	0.05892	0.1772	0.1984	0.1416
Oxidative phosphorylation	0.1615	0.209	1.5E-05	1.21E-06	3.06E-11
Purine metabolism	0.04464	0.007815	5E-10	9.53E-12	0.04657
Pyrimidine metabolism	0.4328	0.4945	1.18E-06	3.93E-09	0.3854
Ribosome	1	0.2057	1.6E-08	8.18E-11	2.2E-16
Sulfur relay system	0.01899	0.02263	0.000106	1.14E-05	1
Vitamin B6 metabolism	0.5726	0.2341	0.005584	0.000695	1

Table S2. Comparison of PseudoCAP functions for the DEGs of *P. aeruginosa* at different infection time point.

PseudoCAP function	Total number	5 min		10 min		20 min		30 min		80 min	
		Up	Down	Up	Down	Up	Down	Up	Down	Up	Down
Adaptation, Protection	179	0	1	0	14	0	51	0	64	0	4
Amino acid biosynthesis and metabolism	239	0	1	0	28	0	103	0	133	0	1
Antibiotic resistance and susceptibility	55	0	0	0	1	0	9	0	14	0	0
Biosynthesis of cofactors, prosthetic groups and carriers	160	0	2	0	11	0	67	0	72	0	0
Carbon compound catabolism	172	0	0	1	9	1	34	3	42	0	0
Cell division	29	0	0	0	1	0	21	0	27	0	0
Cell wall / LPS / capsule	182	0	1	0	17	0	54	0	61	1	0
Central intermediary metabolism	99	0	1	0	4	0	31	0	40	0	0
Chaperones & heat shock proteins	56	0	0	0	3	1	20	0	25	0	2
Chemotaxis	64	0	2	0	9	0	48	0	57	0	2
DNA replication, recombination, modification and repair	88	0	0	0	8	0	33	0	37	0	1
Energy metabolism	206	0	0	0	16	0	74	0	104	0	0
Fatty acid and phospholipid metabolism	62	0	0	0	8	0	27	1	26	0	3
Hypothetical, unclassified, unknown	2001	0	9	5	221	12	528	12	622	0	19
Membrane proteins	675	0	5	2	38	8	149	8	166	0	4
Motility & Attachment	121	0	3	0	20	2	51	0	51	1	1
Non-coding RNA gene	105	0	0	0	0	0	6	1	11	0	1
Nucleotide biosynthesis and metabolism	87	0	0	0	7	0	40	0	47	0	2
Protein secretion/export apparatus	124	0	0	0	6	0	20	0	23	0	1
Putative enzymes	473	0	3	0	38	0	106	1	128	0	0
Related to phage, transposon, or plasmid	65	0	1	1	1	0	6	0	9	0	0
Secreted Factors (toxins, enzymes, alginate)	105	0	0	0	5	0	13	0	17	0	0
Transcription, RNA processing and degradation	55	0	0	0	4	1	30	1	34	0	0
Transcriptional regulators	476	0	8	0	63	1	120	0	126	0	5
Translation, post-translational modification, degradation	197	0	0	0	8	1	98	0	124	0	13
Transport of small molecules	597	0	2	1	36	4	144	5	169	1	4
Two-component regulatory systems	121	0	1	0	13	0	38	0	38	0	3

Table S3. Gene co-expression analysis of phage and host transcriptional regulators.

Time	Host gene	Phage gene	cor	*p*-value	Host gene Function
10min	*vqsR*	ORF50	-0.99029	0.009722	QS system and virulence
10min	*argR*	ORF52	-0.99052	0.009722	Arginine metabolism
10min	*gacA*	ORF53	-0.99956	0.005556	Biofilm
10min	PA2588	ORF53	-0.99481	0.005556	
10min	*rpoS*	ORF53	-0.99507	0.005556	QS system and virulence
10min	*vqsR*	ORF53	-0.99014	0.001389	QS system and virulence
20min	PA1015	ORF28	-0.99148	0.004167	
20min	PA2551	ORF29	-0.99693	0.006944	
20min	PA2551	ORF30	-0.99422	0.006944	
20min	PA2551	ORF32	-0.99353	0.006944	
20min	*gacA*	ORF33	-0.99088	0.001389	Biofilm
20min	*gacA*	ORF34	-0.9987	0.006944	Biofilm
20min	*gacA*	ORF36	-0.99195	0.004167	Biofilm
20min	*gacA*	ORF40	-0.99564	0.004167	Biofilm
20min	*gacA*	ORF44	-0.99715	0.004167	Biofilm
20min	PA2096	ORF45	-0.99096	0.006944	
20min	PA1261	ORF46	-0.99155	0.008333	
20min	PA2096	ORF46	-0.99331	0.008333	
30min	PA1261	ORF19	-0.99117	0.005556	
30min	*argR*	ORF23	-0.99053	0.001389	Arginine metabolism
30min	PA2096	ORF27	-0.99493	0.008333	
30min	PA4878	ORF31	-0.99348	0.005556	
30min	PA1859	ORF39	-0.99304	0.008333	
30min	PA2588	ORF39	-0.99609	0.002778	
30min	PA0163	ORF52	0.993627	0.009722	

Table S7. Primers for Real-Time qPCR.

16S rRNA-F	CAAAACTACTGAGCTAGAGTACG
16S rRNA-R	TAAGATCTCAAGGATCCCAACGGCT
rpsC-F	ACGCAGACCGCAAGAACTATG
rpsC-R	GCACGGGCACACCCATTTG
rpsD-F	GACCGTCAACATCCCTTCCTACC
rpsD-R	GAGTTCCAGAGCCTGAGCGATAC
rplN-F	GGTCGTTCGCACCAAGCAC
rplN-R	ACTTCTCGGTACGGAGTTCACG
pdxH-F	GCGATCCCTTCAGCCTGTTC
pdxH-R	CTTGGCGCTTTCGTAGTTGGT
dadA-F	GCTACCGCAACCTGTTCCTCA
dadA-R	GGCATTCTTGGCGTTCTCC
ndh-F	ACGCCAACCTCACCCACAT
ndh-R	CGCCATTCTCGTCCACCA
rpoS-F	CGATCATCCGCTTCCGACCAG
rpoS-R	CTCCCCGGGCAACTCCAAAAG
fabA-F	CCGGGTAACGCGCAACT
fabA-R	CCGACATCGCTGATGTGAAC
